# Supplementary material for: Combined Effect of Genotype, Housing System, and Calcium on Performance and Eggshell Quality of Laying Hens
Source: Animals (Basel). 2020 Nov 16;10(11):2120. doi: 10.3390/ani10112120 (PMC7696842; doi:10.3390/ani10112120)
Supplement: Supplementary file 1 [file animals-10-02120-s001.pdf]

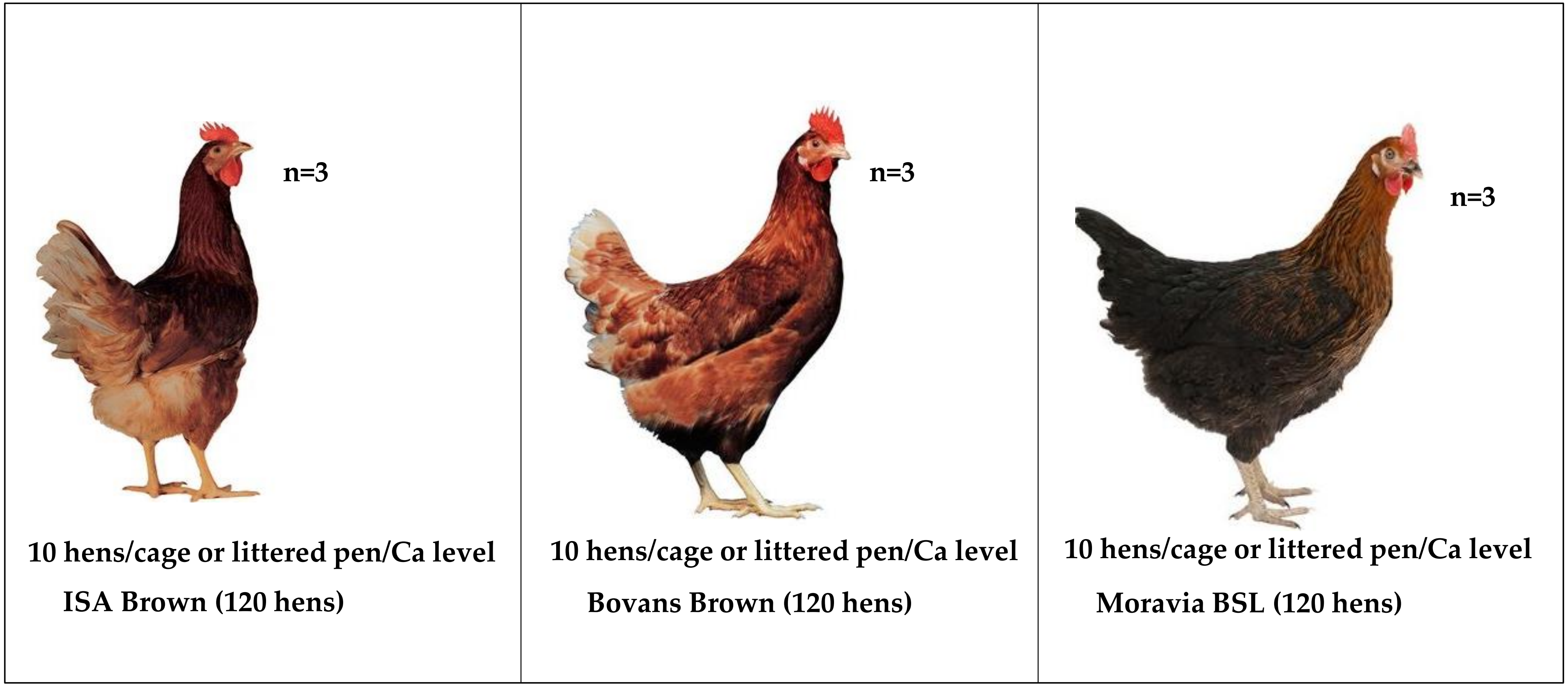


**Figure 1.** Visual representation of the experimental design.
